# Analyzing spatial patterns linked to the ecology of herbivores and their natural enemies in the soil

**DOI:** 10.3389/fpls.2013.00378

**Published:** 2013-09-30

**Authors:** R. Campos-Herrera, J. G. Ali, B. M. Diaz, L. W. Duncan

**Affiliations:** ^1^Departamento de Contaminación Ambiental, Instituto de Ciencias Agrarias, Consejo Superior de Investigaciones CientíficasMadrid, Spain; ^2^Entomology and Nematology Department, Citrus Research and Education Center, University of FloridaLake Alfred, FL, USA; ^3^Department of Ecology and Evolutionary Biology, Cornell UniversityIthaca, NY, USA; ^4^Departamento de Protección Vegetal, Instituto de Ciencias Agrarias, Consejo Superior de Investigaciones CientíficasMadrid, Spain

**Keywords:** PCR-based molecular methods, soil food webs, herbivore-induced plant volatiles, SADIE analysis, biological control

## Abstract

Modern agricultural systems can benefit from the application of concepts and models from applied ecology. When understood, multitrophic interactions among plants, pests, diseases and their natural enemies can be exploited to increase crop production and reduce undesirable environmental impacts. Although the understanding of subterranean ecology is rudimentary compared to the perspective aboveground, technologies today vastly reduce traditional obstacles to studying cryptic communities. Here we emphasize advantages to integrating as much as possible the use of these methods in order to leverage the information gained from studying communities of soil organisms. PCR-based approaches to identify and quantify species (real time qPCR and next generation sequencing) greatly expand the ability to investigate food web interactions because there is less need for wide taxonomic expertise within research programs. Improved methods to capture and measure volatiles in the soil atmosphere *in situ* make it possible to detect and study chemical cues that are critical to communication across trophic levels. The application of SADIE to directly assess rather than infer spatial patterns in belowground agroecosystems has improved the ability to characterize relationships between organisms in space and time. We review selected methodology and use of these tools and describe some of the ways they were integrated to study soil food webs in Florida citrus orchards with the goal of developing new biocontrol approaches.

## Introduction

Challenges for modern agriculture include producing enough food while simultaneously reducing negative impacts on the environment and using our resources in sustainable ways. A major challenge in ecology is understanding where multitrophic interactions unfold, and how to characterize and interpret them. An objective common to both realms is the development of a more holistic understanding of interacting organisms that affect plants directly and through ancillary processes such as soil fertility and levels of pests and diseases. An additional aspect consistent in both agriculture and ecology is that plant relationships with members of the belowground community surrounding roots receive far less attention than their aboveground counterparts (Hunter, 2001). Although they are an undoubtedly important and vital facet of plant health, these rhizosphere communities are often overlooked either by convention or because of the difficultly associated with observing, processing, and quantifying cryptic organisms. By identifying organisms that contribute to the complex multitrophic interactions in the soil it becomes possible to infer the existence of underlying processes, such as intraspecific (reproduction, dispersal, mortality) and interspecific (competition, predation) interactions among organisms or responses to environmental heterogeneity (Perry and Dixon, 2002; Ings et al., 2009). Such research allows for advances in both sustainable agricultural practices and the foundation of plant-based interactions in ecology.

Recent methodological advances in biology, chemistry, and statistics have resulted in unprecedented opportunities in agroecology. The growing availability of molecular genetics methods, public datasets, and free software via online platforms has fostered the use of PCR-based techniques for accurately exploring the diversity of organisms, their relationships, and their functions in ecosystems. Advances in chemical ecology techniques are facilitating the study of belowground signals and cues which play intricate roles between organisms and draw linkages between species and their responses to one another across trophic levels. The characterization and analysis of the spatial patterns of organisms involved in chemical-mediated interactions is one approach to identifying their linkages with one another and with physical attributes of the soil environment. These areas when overlaid allow for the quantification of cryptic organisms and the interpretation of communication between them in different environments.

In this paper, we shall consider selected approaches that can facilitate studies of the subterranean plant environment and emphasize how they can be integrated to enhance biological control of crop pests (Ali et al., 2013; Campos-Herrera et al., 2013). The use of molecular tools to identify and quantify organisms at multiple trophic levels is providing opportunities to characterize targeted species in complex and cryptic soil systems with precision (Campos-Herrera et al., 2011a,b, 2012). Next generation sequencing (NGS) systems and high-throughput tools can reveal previously unknown organisms and novel functions of soil communities (He et al., 2010; Hackl et al., 2012). In a similar way, soil organisms perceive the environment and communicate with each other by using different physical and chemical signals (Torr et al., 2004; Rasmann et al., 2005; Dillman et al., 2012). In particular, chemical cues soluble in the soil matrix are considered of great interest for the biocontrol potential use in agroecosystems. Improved methods to more accurately characterize flux in mixtures of volatile gases in the soil pores presents the opportunity to understand semiochemical signaling that regulates interactions between plants and organisms in their rhizospheres (Ali et al., 2012, 2013; Robert et al., 2013). Spatial and temporal linkages between soil organisms and their relationships with the abiotic environment are most commonly made with multivariate statistical or geostatistical methods. Spatial analysis by distance indices (SADIE) is a less used method, described here because it uniquely employs spatial and temporal data in a way that also provides fiducial limits to inferences regarding spatial aggregation and the spatial or temporal association between variables. We conclude the paper with an example of how these methods are being used to study how soil food webs might modulate the rates of an herbivore-disease complex across different habitats where citrus is grown in Florida.

## Molecular techniques for characterizing soil communities and food webs

PCR-based techniques are employed to identify and quantify numerous organisms in complex matrices such as soil, gut content or fecal samples. They are also used with RNA to characterize gene expression in communities (e.g., Mengoni et al., 2005), an application reviewed by Bustin et al. (2009) and van Pelt-Verkuil et al. (2010). Because the same methods are employed to measure all targeted organisms, these techniques have greatly extended our ability to study cryptic species, associations and processes in nature (Carreón-Martínez and Heath, 2010; Campos-Herrera et al., 2011b). PCR methods are used routinely to accurately monitor bacteria, fungi, nematodes, insects, and associated cryptic organisms (Hoogendoorn and Heimpel, 2001; Atkins et al., 2005; Gariepy et al., 2007; Wang et al., 2009; Campos-Herrera et al., 2011b; Griffiths et al., 2012; Pathak et al., 2012; Hilton et al., 2013). They are an important alternative to traditional morphological identification, especially in their ability to detect and identify juvenile stages, very small quantities of tissues and even degraded material (Chen et al., 2010; Pathak et al., 2012). The limited taxonomic expertise required is especially important for studies of complex food webs involving organisms at several trophic levels. PCR-based methods also permit direct measurement of interacting organisms, eliminating the need for artificial culturing that can confound population estimates (Chen et al., 2010; Pathak et al., 2012).

Following are descriptions of some PCR-based methods being used to study subterranean communities, along with examples used to illustrate their comparative advantages or disadvantages.

### PCR-based methods used for belowground studies in agroecosystems

Aboveground predator-prey interactions were first studied with PCR-based methods in the early part of this century. These studies employed conventional PCR protocols to investigate interactions between diverse groups such scarabids and slugs (Dodd et al., 2003) and spiders, hemipterans and lepidopterans (Ma et al., 2005). The methods soon evolved to permit the use of several specific primers simultaneously in the same multiplex reaction (Agustí et al., 2003; Juen and Traugott, 2006, 2007; Traugott et al., 2006). For example, several belowground invertebrates were identified by multiplex PCR as members of a guild that preys on the garden chafer *Phyllopertha horticola* (Coleoptera, Scarabaeidae) (Juen and Traugott, 2007). Chafer content in the predator gut was detectable for up to 24 h post-feeding. Chafer eggs and larvae served as food for these predators, but appeared to be secondary resources. Although conventional PCR protocols have revealed important insights into ecological interactions aboveground in agroecosystems (e.g., Gagnon et al., 2011; Hatteland et al., 2011; Pumariño et al., 2011; Moreno-Ripoll et al., 2012; Romeu-Dalmau et al., 2012), new techniques such as quantitative real time PCR (qPCR) and NGS are naturally displacing the use of conventional PCR-based methods.

The application of PCR techniques to belowground systems required the development of protocols and systems that separated the target organisms from the soil matrix and avoided the co-extraction of certain chemical compounds such as humic acids that can confound with the PCR reaction (van Pelt-Verkuil et al., 2010). Early studies of belowground community structure used denaturing/thermal gradient gel electrophoresis (PCR-DGGE/TGGE) and terminal restriction fragment length polymorphism (PCR-TRFLP). Both techniques have technical similarities and the common objective of detecting differences in DNA sequences without requiring *a priori* sequencing or background information. They use gel resolution to assess differences among samples. Both amplify the target area using primers complementary to the flanking region (universal or more specific). They depict the biodiversity in a sample by producing a profile pattern of target organisms. Nematode, fungal and bacterial community structures in agroecosystems have been characterized using these techniques (Hagn et al., 2003; Nunan et al., 2005; Sato and Toyota, 2006; Dickie and FitzJohn, 2007; Donn et al., 2007; Wang et al., 2009; Griffiths et al., 2012; Bissett et al., 2013; Hilton et al., 2013) (Table 1). The main advantage of TRFLP with respect the DGGE/TGGE technique is the opportunity to compare data from different runs, whereas cryptic bands and intraspecific variation make the comparison of results between gels difficult (Nunan et al., 2005; Pompanon et al., 2012); however, an important advantage of DGGE is the ability to excise and sequence gel fragments. In addition to these methods, conventional PCR-based methods have also been proposed to study belowground predator-prey interactions (Read et al., 2006; Waldner et al., 2013; Wallinger et al., 2013) including protocols for multiplexing (Harper et al., 2005; Eitzinger and Traugott, 2011). However, as in aboveground systems, the use of qPCR and NGS are replacing these previous techniques.

**Table 1:** **Examples of belowground studies using PCR-based methods for agricultural systems**.

**Agricultural area/field experiment/material employed**	**Ecological measurement**	**Organisms involved**	**PCR-based method**	**References**
Winter wheat in an arable field (Germany)	Community structure	Fungi	DGGE	Hagn et al., 2003
Grazed grassland (UK)	Community structure	Bacteria	TRFLP and DGGE	Nunan et al., 2005
Arable soil (UK)	Community structure	Nematode	Clone and sequencing	Griffiths et al., 2006
Grassland (Kansas, USA)	Community structure	Free-living bacterivorous nematodes	Multiplex qPCR	Jones et al., 2006a,b
—	Community structure	Nematode	DGGE	Sato and Toyota, 2006
Arable soil, dune sand, coniferous forest, pasture, and moorland (UK)	Community structure	Nematode	TRFLP	Donn et al., 2007
Maize crops (USA)	Predator-prey	Insects	qPCR	Lundgren et al., 2009
Natural restoration and cropping management (China)	Community structure	Bacteria	DGGE	Wang et al., 2009
Grazer pasture and forest (South Carolina, USA)	Community structure	Bacteria	Clone and sequencing	Hamilton et al., 2009
Agroecosystems	Predator-prey	Beetles as predators; earthworms as prey	qPCR	King et al., 2010
Agroecosystems	Predator-prey	Mite as predator; nematode as prey	qPCR	Heidemann et al., 2011
Maize crops (USA)	Community structure	Insects (and plant damage linked)		Lundgren and Fergen, 2011
Citrus groves (Florida, USA)	Predator-prey; food webs	Entomopathogenic nematodes, nematophagous fungi, ectoparasitic bacteria, free-living nematodes	qPCR and nested	Campos-Herrera et al., 2011b, 2012; Pathak et al., 2012
Tillage trial (UK)	Community structure	Nematode	d-TRFLP	Griffiths et al., 2012
Agricultural field (Japan)	Community structure	Nematode	Next generation sequencing	Morise et al., 2012
Abandon field (Netherlands)	Community structure	Nematode	qPCR	Vervoort et al., 2012
Tillage and nutrient additions in wheat cropping experiment (Australia)	Community structure	Bacteria	TRFLP	Bissett et al., 2013
Oilseed rape fiel trial (UK)	Community structure	Fungi and bacteria	TRFLP	Hilton et al., 2013

Note that we have excluded references corresponding to the development of new molecular methods for particular species, without establishing relationship among other (structure or food web).

Quantitative real-time PCR (qPCR) has been extensively developed for belowground systems (Table 1). As with conventional PCR protocols, qPCR uses species-specific molecular markers to identify and quantify target species. The advantage of qPCR compared to conventional PCR is that the order of magnitude increase in the amplification potential of tiny quantities or even degraded material. This method has been successfully employed to detect cryptic organisms and assessed diversity and interactions among bacteria, fungi, nematodes, insect and plants from above and belowground systems (Atkins et al., 2005; Jones et al., 2006a,b; Zhang et al., 2006; Lundgren et al., 2009; King et al., 2010; Campos-Herrera et al., 2011a,b, 2012; Heidemann et al., 2011; Lundgren and Fergen, 2011; Pathak et al., 2012; Vervoort et al., 2012). Nevertheless, qPCR also has important limitations. For example, not all species contain sequences in the genes currently used to discriminate taxa that are suitable for valid primers, or in some cases probes. Some groups, such as free living nematodes, frequently lack adequate numbers of published sequences for comparison when designing molecular probes, so this method can finally be used for some undescribed, regional species (Jones et al., 2006a,b). In such cases, it may still be useful to use qPCR to measure broader taxa with similar ancestry/behavior rather than individual species (Campos-Herrera et al., 2012). The worldwide tendency to increasingly publish sequences in public domains is rapidly increasing the opportunity to design species-specific primers/probe combinations for a diverse array of organisms. Another current issue for qPCR technology is the reproducibility and viability of multiplexing in order to reduce the expense of these protocols. Often, the use of several species-specific markers in the same reaction reduces the amplification of target organisms if the initial quantities are minimal. Although some multiplex reaction have been described (Jones et al., 2006a; Berry et al., 2008), it is advisable to perform single reactions for precise estimation because competition between target DNAs or development of interacting primers (primer-dimers) can impede or produce a false signal (Zijlstra and van Hoof, 2006; Berry et al., 2008). This approach is particularly appropriate when evaluating samples with quarantine requirements.

DNA barcoding is a PCR-based method used extensively in studies of belowground systems, especially to assess community structure (Table 1). Two main approaches have been used in the last decade: clone-sequencing (i.e., insert the PCR product, usually in a plasmid, and get the sequence) and NGS, also called high-throughput sequencing, which does not require cloning. These two techniques obtain the full sequence of the products derived with selected primers and both are quantitative and species specific. Barcoding approaches are rapidly expanding the ability to study concomitant organisms in soil samples, for example, to compare community structure under different management programs. Important limitations include high reaction and equipment costs (that are rapidly declining) and the complexity of the amplicons that are generated. A great deal of time and experience are required to organize and assess the validity of hundreds or thousands of generated amplicons. Increasingly, new bioinformatic skills are used as substitutes for zoological-taxonomical expertise in order to provide an ecological context for the molecular operational taxonomic units (MOTUs) that are identified from multiple-alignments and classified by their associations with one another (Blaxter et al., 2005). Of course, if taxa defined by MOTU methods are used for ecological purposes, they must eventually be associated with sequences from specimens identified by Linnaean taxonomy.

Other new PCR-based systems emerging during the last 5 years are some that use microfluidic systems (also microfluidic droplet PCR) (Zhu et al., 2012; Chang et al., 2013). Although these new protocols were developed for human clinical diagnostics, their application in agroecological studies is only a question of time. This technology incorporate chips with microfluidic technology that allows nucleic acid amplification in a compartmentalized reaction in a minimal volume. Their critical advantages are reduced PCR processing time and reaction volume, and substantially reduced cost (Zhu et al., 2012; Chang et al., 2013).

### Fundamentals of developing qPCR, DNA barcoding and next generation sequencing protocols

The specificity of qPCR is a consequence of the species-specific primers (and probes, if used) that are developed for target species. The biochemistry involves linking fluorescent products to double stranded DNA (e.g., SYBR Green®) or designing specific fluorescent probes (e.g., TaqMan® probes) that provide greater specificity to the primers. Both systems produce increased fluorescence that can be quantified during PCR cycling when used with the targeted species.

In the case of NGS, there are international efforts to define target areas for barcoding standards (i.e., CboL, http://www.barcodeoflife.org); however, exceptions may be desirable depending on objectives (Valentini et al., 2009; Pompanon et al., 2012). The clone-sequencing method has provided outstanding results in studies of distribution, diversity and community structure (i.e., Griffiths et al., 2006; Hamilton et al., 2009), but the technique is more time consuming and costly than new methods of direct amplifications in NGS (Pompanon et al., 2012; van der Heijden and Wagg, 2013). Extensively used NGS platforms include: 454 GS FLX (Life Sciences, Roche), HiSeq 2000 by Illumina (Solexa Technology), and AB SOLiDv4 (Agencourt technology). Each technology is based on a different sequencing approach: 454 GS FLX uses pyrosequencing, HiSeq 2000 sequences by synthesis and AB SOLidv4 by ligation and two-base coding, and all of these methods produce reads of >98% accuracy (Liu et al., 2012). The maximum output of data is generated by HiSeq 2000 (600 Gb) and also is compatible with reads until 3 G for the cheapest cost, estimated as $0.07 per million bases. However, 454 GS FLX has the advantage of a fast read time and can run in just 24 h (Liu et al., 2012). Therefore, the selection of the optimum platform depends on the experimental conditions and requirements (see Glenn, 2011 and Liu et al., 2012).

Several concepts need to be considered when PCR-based experiments are designed for a new organism or taxonomic group. Because real time qPCR and DNA barcoding using NGS are currently the most widely used methods for the study of belowground interactions in agroecosystems, important differences in the requirements for both methods are summarized in Table 2. Technical aspects about the development of these protocols were extensively detailed in Bustin et al. (2009); Campos-Herrera et al. (2010); van Pelt-Verkuil et al. (2010) and Pompanon et al. (2012). The first step is selecting the most appropriate method to address the question. Both techniques require relatively costly reagents. By contrast, while many institutions are able to invest in qPCR equipment used in individual programs, NGS facilities are much more expensive and tend to be supported by multiple users in an institution. The type of data generated by both systems is different, although both can be perfectly complementary. Real time qPCR can detect and quantify (expressed as numbers, copies, quantities, depending on the standard curve units) target organisms accurately. The limitation of this method is that you only find what you are looking for, so it is not possible to detect species for which the appropriate primers (and probes) have not been designed and used. On the other hand, NGS systems provide all the sequences derived from primers selected to amplify a target taxonomic group. Multiple sequences, MOTUs, are then identified that correspond to known and unknown species, in theory. NGS approaches require special attention to avoid “chimera” sequences. Some tools such as QIIME can analyze the data while detecting chimera sequences (Caporaso et al., 2010). If the effects of different agroecosystems on the biodiversity of a target group are of interest, NGS approaches can provide meaningful information. They are also useful when there is no available information regarding the key players in an agroecosystem (Pompanon et al., 2012). However, when there are limited numbers of known organisms interacting in a somewhat predictable manner, qPCR protocols can produce the relevant data in a short time and at the least cost.

**Table 2:** **General considerations for the development of PCR-based methods (from Bustin et al., 2009; Campos-Herrera et al., 2010; van Pelt-Verkuil et al., 2010; Pompanon et al., 2012) and comparison between the two most extended methods in belowground studies: real time qPCR and DNA barcoding using next generation sequencing**.

**Experimental steps**	**Concept**	**Common considerations**	**Real time qPCR**	**Next generation sequencing**
Design “*in silico*”	Selection of the target sequence	Adequate and meaningful for the study; check availability in the system to be able to compare with known species	ITS rDNA, D2D3, COI	SSU, LSU, sometimes ITS, customized
	Coverage and resolution	Range of taxa susceptible to be amplified	Species-specific	Generalistic, amplify broad taxonomic groups
	Primer and amplicon equilibrium	The length and composition of the primers will affect the specificity of the amplification; secondary structures should be avoided; the PCR product, the amplicon, should be into the range for optimal amplification	80–250 bp	200–600 bp, depending on the plataform
	Amplification efficiency	The efficiency might depend on the quality of the DNA (degradated, inhibitors presented, etc.)	Might be improved by adding some reactives (i.e., BSA or DMSO) or by diluting the DNA	
Sample preparation	Design and sampling strategy	Include biological and technical replicates; tagging and multiplexing approaches available	Different dyes for multiplexing	Different molecular tags to separate treatments
	DNA extraction	Multiple kits available; desirable, verify the quality and quantity by electrophoresis or spectrophotometric systems (nano-drop)	–	–
Optimize reaction	PCR conditions	Experimental establishment of annealing temperature, time for extension, number of cycles; check for possible inhibitors	Important the number of cycles in nested qPCR experiments	–
	Sensitivity and specificity	Check the lowest number of amplicons detected of the target species/taxonomic group (dynamic range)	Important for quantification. Serial dilutions of the target DNA will serve for defining the limit of accurate detection for our standard curve	Important to establish the minimum taxonomic unit detected
Data analysis and validation of the experiments	Type of generated data	Units or type of quantification	Detection and quantification of the target organisms. Absolute quantification is possible if a standard curve is included in the run; relative quantification is possible among target species	Molecular operational taxonomic units (MOTUs). Special care need to be taken for the detection of “chimera” sequences, as a subproduct of amplification that provide a non real sequence
	Taxa assignation	Identification with species or taxonomic group with known identity and possible defined ecological traits	Amplifications are compared with the positive control, the DNA from the known target organisms; additionally, postsequencing analysis can be performed and comparison with reference database	Comparison with reference database (i.e., GenBank, IBOL, EMBL, DDBJ or customized for specific studies)
	Repeatability, reproducibility, and accuracy	Measurement of the intra-assay variance, inter-assay variance and difference between experimental measurement and actual values, respectively	Critical to compare measurements from a run to another	Desirable, although costly

Although molecular methods are alternatives to conventional methods that rely on morphological identification, they should often complement rather than replace traditional methods. For example, Campos-Herrera et al. (2011a) found a strong positive correlation between known quantities of nematodes and numbers measure by qPCR, whereas Griffiths et al. (2006) suggest that molecular techniques underestimated certain groups compared to observations using morphology. Gibb et al. (2008) suggested that congruence of morphology and molecular characterization can be confounded by steps that reduce the recovery efficiency or the PCR amplification. The study of the arbuscular mycorrhizal fungi (AMF) provides another good example of why both approaches complement each other, because the biological species concept of AMF is still unresolved (Sharmah et al., 2010).

## Methods for detecting herbivore-induced cues belowground

Plant volatile organic compounds (VOCs), which mainly comprise terpenoids, fatty acid derivatives, phenylpropanoids and benzenoids (Dudareva et al., 2004) have been the center of intensive studies of plant-herbivore-predator interactions for more than two decades (Dicke and Sabelis, 1988; Turlings et al., 1990). VOCs blends can be complex, comprising hundreds of compounds, some of which are not produced by intact or mechanically damaged plants but are synthesized *de novo* in response to herbivore attack (Turlings and Wäckers, 2004; Mumm and Dicke, 2010). Indirect defense is described as a phenomenon of plant defense strategy where plants attract enemies of the herbivore when “attacked” (Dicke and Sabelis, 1988; Turlings et al., 1990). Although a diversity of root derived exudates can play a role in the rhizoshpere community (Badri and Vivanco, 2009), root volatiles can and have recently been shown to play a role in contexts that might change the abundance, location, and diversity of organisms in the soil in a manner similar to signals aboveground (Ali et al., 2013).

Research on plant VOCs produced after insect herbivory has been dominated by studies aboveground (see Kessler and Morrell, 2010), probably due to methodological constraints related to subterranean investigations. More recently, the number of studies showing that herbivore induced belowground volatiles trigger predator attraction in the soil has increased. Belowground investigations began with two key studies which demonstrated for the first time that unknown cues were responsible for attracting entomopathogenic nematodes to insect damaged roots (Boff et al., 2001; van Tol et al., 2001). Tritrophic interactions with belowground herbivore-induced plant volatiles (HIPV) signaling have been described both in agricultural systems (Rasmann et al., 2005; Ali et al., 2010, 2011) and in a natural context (Rasmann et al., 2011). Because of the extensive literature focused on aboveground plant volatiles and methods, here we describe the techniques used for belowground assessment, focusing our attention on the advances related to agroecosystems and their application in the control of insect pests.

Factors in addition to VOCs are known to influence both behavior of herbivores and their natural enemies belowground. Root exudates also influence spatial distribution of root feeders (Johnson and Gregory, 2006; Johnson and Nielsen, 2012; Gfeller et al., 2013). Some recent techniques were developed to measure root exudates that influence herbivore behavior using LESA (liquid extraction surface analysis) (Robert et al., 2013). Although these studies are sound and contribute to belowground interactions we shall focus here on cues that have been used in agricultural/biological control contexts and have been detected with non-destructive techniques, sampling/manipulating behavior in a manner that allows for large scale patterns in field research. Thus, far VOCs have been a major candidate for use in biological control and also to explain multitrophic cascades both above and below ground (Kaplan, 2012a,b; Poelman et al., 2012; Ali et al., 2013).

### Methods for detecting belowground herbivore-induced volatiles

The technical difficulties associated with dynamics of the soil ecosystem have been a major limitation in studying belowground multitrophic interactions. Soil is a complex, tri-phasic medium making the analysis of individual factors and their interactions considerably difficult, and hence, most research has been based on *in vitro* analysis of individual factors (Rasmann et al., 2012a). Researchers willing to study factors associated with roots signals are often focused on the study of (i) root diffusates (often used to convey non-volatile substances diffusing through the soil and establishing a gradient), (ii) root leachates (method of obtaining an extract from the roots, more than it does to the solution itself), and or (iii) root exudates (most often restricted to liquids that gradually “ooze” from its source, but can be applied to volatiles as well). New approaches to evaluate root volatiles have only recently been developed and applied in researches on chemical and evolutionary ecology. As mentioned earlier, all of these can serve as signals and cues for herbivores and their natural enemies. However, in this review we focus on HIPVs as they are often shown to be involved in multitrophic interactions that include natural enemies and have been shown to be detectable from intact plants. Although methods exists for the evaluation of additional exudates from intact plant roots (i.e., polydimethylsiloxane (PDMS) for Solid phase root zone extraction (SPRE), see Mohney et al., 2009), thus far they have not been used to evaluate induction related to root herbivores.

Rasmann et al. (2005) provided for the first time the evaluation and identification of indirect volatile defenses of maize roots using solid phase micro-extraction (SPME), which is a method of sampling volatiles without the use of solvents. Briefly, an adsorbent-coated fused silica fiber (with properties similar to a gas chromatography column) can collect volatile compounds from the headspace of a sample. The volatile compounds once fixed to the SPME fiber can then be thermally desorbed in an injection port of a gas chromatograph. Then, these can be further analyzed and/or identified when coupled with known standards or libraries of mass spectroscopy. Rasmann et al. (2005) crushed flash-frozen roots samples, either fed-upon or non-fed-upon into a fine powder to analyse the effects root herbivory had on the plant produced VOCs. This powder was then exposed to the SPME fiber, allowing all the volatiles that had accumulated in either treatment to be sampled and compared with Gas Chromatography-Mass Spectrometry (GC-MS). SPME is a rapid and simple extraction method that does not require the use of solvents and its detection limits can reach parts per trillion (ppt) levels for certain compounds (Pawliszyn, 1997). However, one of the limitations of this technique is that it is a destructive method of sampling root material: both the plant and herbivore must be separated and volatiles from this interaction can only be examined after harvesting and crushing the plant tissues.

**Figure 1:**
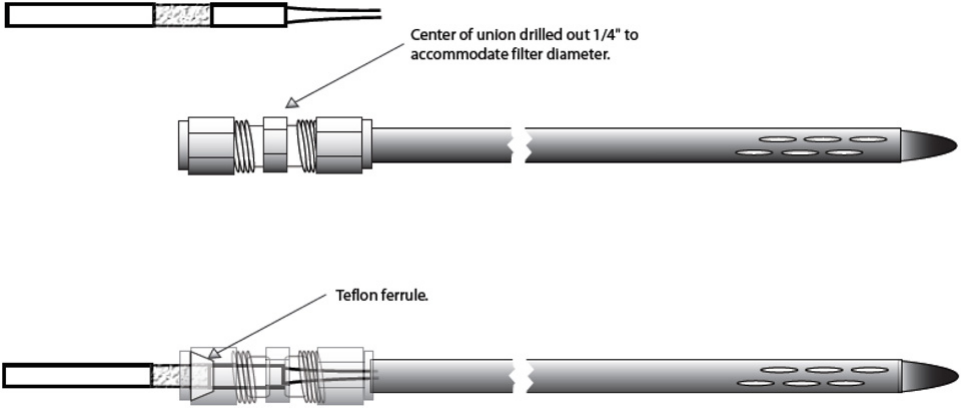
**Representation of soil probe design used to sample volatiles belowground**. Probe is inserted into soil and connected to a vacuum pump. Reprint from “Extending explorations of belowground herbivore-induced plant volatiles: attracting natural enemies of root pests in multiple contexts (Ali et al., 2012).

Ali et al. (2010) were able to non-destructively sample belowground herbivore induced volatiles from citrus roots using another technique, a flow-through dynamic sampling coupled with adsorbent traps. In this case, volatiles can be collected and extracted by elution of an adsorbent with low boiling point solvents. Adsorbent traps are usually made of glass tubes filled with the granulated adsorbent, held in place by stainless steel mesh, glass wool plugs, or Teflon fitted rings. By connecting the adsorbent trap to a vacuum pump and pulling air through glass chambers (containing intact citrus plants either with or without feeding larvae) Ali et al. (2010) were able to sample volatiles associated with belowground herbivory *in situ* using a non-destructive approach. The volatiles collected on this trap are rinsed using a non-polar (e.g., methanol, hexane, dichloromethane) solvent and analyzed with GC-MS. This methodology allows for the sample to be retained in a solvent, which can be analyzed multiples times. Additionally, the solvents containing root volatiles were also tested in sand-filled two-choice bioassays chambers. By using this approach, Ali et al. (2010, 2011) found evidence for entomopathogenic nematode attraction to volatiles from infested citrus roots. Moreover, a soil probe was recently used to sample soil volatiles in Florida citrus groves at a depth of 20 cm (Figure 1) (Ali et al., 2012), which constitute an important advance in the study of volatiles *in situ.* This soil probe was developed to collect the compound on adsorbent traps by using a vacuum pump to pull air from the soil. Recently, a non-destructive method similar to the one used by Ali et al. (2010) to collect root volatiles was also successfully used to detect (*E*)-β-caryophyllene from maize roots (Robert et al., 2013).

Proton-Transfer-Reaction Mass-Spectrometry (PTR-MS) is an additional non-destructive analysis that has recently been applied to the detection of belowground trace HIPVS in real-time (Danner et al., 2012). Briefly, the PTR-MS technology pumps air to be analyzed continuously through a drift tube reactor, and a fraction of the VOCs is ionized in proton-transfer reactions with hydronium ions (H_3_O^+^). The ionized molecules usually form a protonated molecular ion [M+H]^+^, where “M” is the molecular mass of the parent molecule (for detailed descriptions of the PTR-MS technology see Hansel et al., 1995; de Gouw et al., 2003; Boamfa et al., 2004). By using this technique, organic compounds such as ketones, aldehydes, alcohols, oxygenated aromatic and aliphatic compounds will be readily protonated (Danner et al., 2012). Proton transfer utilizes a soft ionization method, so it generally leads to less overall fragmentation of the product ions, which is a tremendous advantage, and also the mass of the product ion equals the VOC mass plus one (de Gouw and Warneke, 2007; Danner et al., 2012). At the end of the drift tube, the reagent and product ions are measured by a quadrupole mass spectrometer. The product ion signal is proportional to the VOC mixing ratio. PTR-MS can monitorize numerous VOCs with a high sensitivity (10–100 pptv) and rapid response time (1–10 s) (de Gouw and Warneke, 2007), although regular calibration with the gas mixture is required for accurate and reproducible quantification of trace gases (Danner et al., 2012).

There are many advantages of the PTR-MS method. The more common methods of collecting plant VOCs on to filters with polymer adsorbents makes it necessary to preconcentrate the sample before analysis by collecting volatiles during a range of minutes to hours; this reduces the ability to resolve the timing of VOC emission measurements (Danner et al., 2012). Furthermore, solvents used to elute VOCs from the adsorbents may introduce contamination before GC analysis. A major disadvantage is that PTR-MS only determines the mass of product ions, which is, of course, not a unique indicator of the VOC identity. It is clear that isomers cannot be distinguished, and the interpretation of mass spectra is further complicated by the formation of cluster ions and the fragmentation of product ions. A significant experimental effort has therefore been made to characterize the specificity of PTR-MS for different VOCs.

Early studies using PTR-MS already has shown how it can be successfully applied to analyze VOCs produced by aboveground (wounded) plant parts (Fall et al., 1999). Two recent examples show its promise as a technique to evaluate belowground interactions in preliminary PTR-MS results on herbivore-induced root responses in *Brassica* species. Danner et al. (2012) monitored VOCs emanating from roots of potted turnip plants (*Brassica rapa* subsp. *rapa* var. Nancy) during infestation with a belowground herbivore, *Delia radicum*, the larvae of the cabbage root fly. The resulting mass scans found that the intensities of several molecular masses were enhanced in root fly infested *B. rapa* roots (Danner et al., 2012). In a second example, Danner et al. (2012) monitored the induction of VOCs in *Brassica juncea* roots after infestation with *Delia radicum* in real-time and compared it to a control treatment. Initially, they detected only a low emission rate of compounds, which steadily increased with longer feeding times of the root flies. In control plants, the VOC emissions remained at a very low level, allowing a clear distinction between control and infested plants within a few hours after infestation (Danner et al., 2012).

In general, these techniques are informative and effective in different manners. For example, the non-destructive sampling techniques are useful in evaluating belowground interactions *in situ*. Also, they may potentially prove useful in additional contexts. However, the properties of the surrounding soil might interfere and, hence, make resolution difficult with the potential for significant background. In this case, SPME can be considered, since eliminates such background, although it can introduce complications from tissue maceration (enzymes or oxidation can rapidly change the chemical profile) and may not accurately represent the blend released from intact living organisms (Tollsten and Bergström, 1988; Heath and Manukian, 1992). The combination of techniques and refinements of approaches might produce the best resolution for the target individual system.

### Examples of belowground herbivore induced plant volatiles in agroecosystems

The considerable advances in research on molecular mechanisms and ecological signaling of insect herbivore induced VOCs launch promising prospects of manipulating the release of these compounds in order to enhance crop protection. Encouraging examples from both laboratory and field experiments support this approach to develop novel ecologically drive crop protection strategies. On the agricultural side, at the moment, the best-known belowground tritrophic interaction is the maize system, described for the first time by Rasmann et al. (2005). When the larvae of the western corn rootworm, *Diabrotica virgifera virgifera*, attack the roots, European maize varieties emit in soil the sesquiterpene (*E*)-β-caryophyllene (EβC) (Rasmann et al., 2005; Kollner et al., 2008). This compound is a highly attractive HIPV to the entomopathogenic nematode *Heterorhabidtis megidis* in the laboratory as well as in the field (Rasmann et al., 2005; Kollner et al., 2008; Hiltpold et al., 2010a). Several laboratory and field experiments with various corn lines and synthetic compounds have shown that EβC is an ideal compound to diffuse through the complex belowground soil matrix. In fact, it is among the less costly terpenoid that could be travelling within the soil (Hiltpold and Turlings, 2008), and that is under selection (Kollner et al., 2004, 2008).

Another example of highly complex volatile blends in agroecosystems was described by using the roots of cotton (*Gossypium herbaceum*) and the larvae of the chrysomelid beetle *Diabrotica balteata*. After feeding by this generalist root feeder, cotton plants were scored to emit >10 compounds, where at least 7 terpenoid volatiles were observed (Rasmann and Turlings, 2008). The sesquiterpenoid aristolene was discussed as being a good candidate for playing a major role in *Heterorhabditis megidis* nematode attraction, although future studies might confirmed it (Rasmann and Turlings, 2008). These authors also tested the nematode preference against damaged roots of cowpea (*Vigna unguiculata*) plants. In contrast to the other crops, corn and cotton, cowpea plants emitted almost undetectable amounts of volatiles that also resulted in lower nematode attractions (Rasmann and Turlings, 2008).

More recently, Ali et al. (2010) have demonstrated that citrus roots upon feeding by the root weevil *Diaprepes abbreviates* emit several terpenes in the surrounding soil. Using belowground olfactometers Ali et al. (2010, 2011) found that the entomopathogenic nematodes were significantly more attracted to citrus roots infested with the insect larvae than by roots mechanically damaged or pots containing only soil. However, Ali et al. (2011) observed that insect induced roots of citrus tree could also attract the plant-parasitic nematode *Tylenchulus semipenetrans*. Consequently, this may reduce the exploitation of citrus induced VOCs emission in biological control strategies targeting *Diaprepes abbreviates* if rootstocks are not truely resistant to *T. semipenetrans*. Most recently, Ali et al. (2012, 2013) has combined qPCR methods with the field application of root larvae induced volatiles to evaluate attraction of multiple nematode species and nematophagous fungi (NF). This research shows that multiple species of naturally occurring entomopathogenic nematodes as well as “hyperparsites” of entomopathgenic nematode killed cadavers are responding to this cue (Ali et al., 2013).

### Application in agriculture: manipulation and enhancement of belowground signals

At this moment, there are very few published examples of cue-based behavioral manipulation in belowground contexts. The use of natural products to enhance biocontrol is typically compatible with integrated pest management; deploying HIPVs aboveground by controlled release dispensers has been shown to increase plant recruitment and retention of beneficial parasites or predators (Thaler, 1999; James and Grasswitz, 2005). In an analogous belowground investigation, entomopathogenic nematode infection of *Diabrotica virgifera virgifera* larvae was increased by spiking soil surrounding maize roots with the HIPV, EβC (Rasmann et al., 2005). Ali et al. (2012) has recently increased mortality of root pests in the field by enhancing host location of naturally occurring entomopathogenic nematodes in citrus and blueberry crops with the application of the citrus root volatile, 1, 5-dimethylcyclodeca-1, 5, 7-triene (pregeijerene).

After identification of herbivore-induced compound attractive to natural enemies, it is possible genetically manipulate the plant in order to (i) make a plant more attractive to beneficial predators or parasitoids or (ii) to restore the phenotype that was lost due to natural or human selection (Rasmann et al., 2012a). The first examples of such an approach are aboveground (Kappers et al., 2005). More recently, the terpene synthase gene TPS23, which is responsible for the synthesis of Eβ C, has been identified in maize (Kollner et al., 2008). Most of the European maize varieties and Teosinte produce this sesquiterpene whereas American varieties do not (Rasmann et al., 2005; Kollner et al., 2008). This indicates a shift in the gene activity through breeding selection (Kollner et al., 2008). Degenhardt et al. (2009) restored the ability of maize to recruit entomopathogenic nematodes by inserting a TPS23 gene from *Origanum vulgare* into a non-producing maize line, demonstrating in a filed experiment that the transformed maize line was significantly more attractive for the entomopathogenic nematode *Heterorhabditis megidis* compared to the wild type, leading to increased protection for transformed plants. This constituted the first demonstration in the field that plant genotype engineering could enhance biological control.

Entomopathogenic nematodes appear as good candidates for an inundative biological control strategy. At the moment, there are knowledge of key attractants for specific entomopathogenic nematodes species (Hallem et al., 2011). (Hiltpold et al., 2010a) evaluated whether selection for enhanced responsiveness to the crucial root signal EβC could improve the efficiency of these nematodes in controlling the larvae of the chrysomelid beetle *Diabrotica virgifera virgifera*. Using root-zone olfactometers, a population of the nematode *Heterorhabditis bacteriophora* was successfully selected. Originally, this nematode population did not respond to attraction effect by the emision of EβC (Hiltpold et al., 2010b) even though its effectiveness in controlling *D. v. virgifera* larvae is high (Kurzt et al., 2009). After the selection process, the new population responded much better to Eβ C in laboratory experiments and also was able to control the pest in the field significantly better in presence of the belowground signal (Hiltpold et al., 2010b). Interestingly, the establishment and the persistence in the field were not influenced by the selection process (Hiltpold et al., 2010b). Here we see the great potential of selecting beneficial organisms for a better and faster response, which also resulted in higher infection rates. Even with some constraints, such as knowledge of key compound/blends, and the laborious selection process, selecting for specific nematode populations could be combined with selection of more attractive plant genotypes, making biological control of insect pests a success (Rasmann et al., 2012a,b).

Recent advances are showing the potential of belowground organisms to interact with aboveground food webs. In this regard, Pineda et al. (2013) have evaluated the mechanism involved in interactions between a beneficial rhizobacterium (*Pseudomonas fluorescens*) and a parasitoid (*Diaeretiella rapae*) by using the model system *Arabidopsis thaliana-Myzus persicae.* In laboratory studies, they combined ecological, molecular, and chemical approaches to study how the rhizobacterial colonization modified the complex composition of the HIPV. They observed that the compounds produced by the rhizobacteria-aphids-plant treatment negatively influenced the behavior of the aphid-parasitoid when compared with the effect of the blend induced by just aphid-plant. These authors have demonstrated that the non-pathogenic rhizobacteria effect on parasitoid activity is mediated by jasmonic acid pathways that associate with plant volatile production. In contrast, work by Robert et al. (2013) has found that the production of an HIPV which is beneficial to the protection of the plant's roots, was simultaneously found to attract aboveground herbivores. Thus, manipulation of these blends and studies in more natural conditions might provide additional insight on the complex multitrophic interactions occurring above- and belowground, and it should be acknowledged the effects and responses in one subsystem of the plant can have very different roles on other systems.

## Methods to measure spatial structure of organisms in field agroecosystems

During the past several decades, new technologies and spatial statistics offer a number of tools for point pattern analysis, which provide improved detection and characterization of spatial heterogeneity such as gradient or clustering (Perry et al., 2002). Earlier two-dimensional maps of spatial patterns were developed for plants and for relatively immobile organisms (Diggle, 1983). Meanwhile, the spatial information of mobile organisms was restricted to counts in traps at specific locations (Perry, 1995). Initially, the methods used to describe spatial patterns focused on the intensity of aggregation and were based on the relationship between the sample mean and variance (Taylor, 1961; Iwao, 1968). Spatial information provided by these methods was criticized by Perry and Hewiit (1991) for not considering geographic location of each sample unit, and in consequence, these indices could not be used for comparing or mapping the spatial patterns of populations.

Currently available technologies (i.e., such as the Stanford Geostatistical Modeling Software (S-GeMS) (http://sgems.sourceforge.net) or the TerraSeer Space-time Information System (STIS) (which public domain software is: http://www.terraseer.com/products/atlasviewer.html) combine the use of geostatistics, as a statistical procedure that uses sample values and locations simultaneously for characterizing spatial patterns and estimating values at unsampled locations (Clark, 2001) and global positioning systems (GPS) to determine the location of each sample unit (Goovaerts, 1997, 1999). Notwithstanding the utility of geostatistical procedures to characterize spatial patterns, they do not provide tests to assess the statistical significance of the estimated patterns. To overcome this limitation, Perry and Hewiit (1991) developed a new method, SADIE, which uses the spatial information in the sample in ways that permit inference testing in order to make the information more understandable from a theoretical biological perspective. Despite this advantage, SADIE is used infrequently compared to geostatistical methods for community studies. Accordingly, we describe the method here with some examples of its application to agroecology.

### SADIE: basic concept and extensions

SADIE is computed with free software, SADIESshell 1.22 (Kelvin F. Conrad and IACR-Rothamsted, 2001) available for download at http://www.rothamsted.bbsrc.ac.uk./pie/sadie/. The method evolved from a spatial analysis based on a single index to an analysis of count data that are spatially-referenced with two coordinates (*x, y*) that can be irregularly spaced and not necessarily on a grid (Perry, 1995, 1998). The development of new SADIE indices and maps, increased the ability to characterize the spatial information in a sample (Perry et al., 1999) and to test the association or dissociation of spatial patterns from two sets of data (Perry and Dixon, 2002) (see chronological development, a brief description of indices and graphical displays provided by this spatial analysis in Table 3).

**Table 3:** **Summary of the main indices and graphical displays provided by SADIE analysis**.

**SADIE indices**	**Definition**	**Graphical display**	**References**
Distance to regularity (*D*)	Measures the minimum effort that the individuals in the sample would need to expend to move to an arrangement where there was an equal number in each sample unit	Initial and final plot (IAF)	Perry, 1995
Aggregation index (*Ia*)	The ratio of *D* to the mean of the simulated distribution		Perry, 1995, 1998
Cluster indices (*vi* and *vj*)	*vi* measures the degree to which the unit contributes to clustering as a member of a patch	“Red-blue plots” Contour map Vector flow plot Empirical distribution function plot of ranked average outflow/inflow distances (EDF)	Perry et al., 1999
	*vj* measures the degree to which the unit contributes to clustering as a member of a gap		
Local and global association index (*X*)	Local association index is calculated by comparing the cluster index (*vi* or *vj*) for each set of data at the same sample unit	Map of local association and dissociation	Perry and Dixon, 2002
	The global index (*X*) is calculated as a mean of the local indices		

SADIE uses a transportation algorithm to move individuals from their initial positions to new ones, using the minimal possible total distance or “distance to regularity” (*D*), in order to convert a given spatial pattern to one of regularity (Perry, 1995). The flows of individuals from areas of relatively high population density to areas where population density is relatively low could be visual observed using an “initial and final plot” (IAF) (Perry, 1995). To assess the magnitude of aggregation, the observed *D* is compared to those for large numbers of randomly distributed permutations of the counts observed among the sample units. The ratio of the observed *D* to the mean of *D*s from the simulated distributions provides an “index of aggregation” (*Ia*). A value of *Ia* = 1 suggests a spatially random pattern, *Ia* > 1 suggests an aggregated pattern and *Ia* < 1 indicates a regular pattern (Perry, 1998). A significance test (*P*a) for the probability that the observed data is no more aggregated than expected from a random permutation of the counts is provided by determining the proportion of simulated *D*s with values less than that of the observed *D* (Perry, 1998).

Perry et al. (1999) described two forms in which aggregation of count data occur: (i) as patch clusters when there is a high density of counts near to one another or (ii) as gap clusters comprising of relatively small or zero neighboring counts. The SADIE analysis provides a local index of clustering (*vi* or *vj*) for each sample point, by employing randomizations in which the observed counts are permutated amongst the sample units. Sample units with counts greater than the overall mean are assigned a patch cluster index (*vi*), which by convention is positive; while units with counts less than the mean are assigned a gap cluster index (*vj*), which by convention is negative. Additionally, SADIE provides for patches an overall index *vi* and associated probability *P*i and for gaps an overall index *vj* and associated probability *P*j. Heuristic threshold fixed values of 1.5 and −1.5 for *vi* and *vj*, respectively, discriminate sampling units associated with index values >1.5 (patches) from sampling units with index values <1.5 (gaps) (Perry et al., 1999). Each local clustering index (*vi* and *vj*) may be contoured by interpolation and mapped using the “red-blue plot” methodology, which enables the characterization of clusters with respect to type (patch or gap), number, position and size (i.e., % or m^2^ of each cluster type) for a given site (Perry et al., 1999). For convention, patches are represented on maps by contoured areas shaded red, where all neighboring sample units have cluster indices *vi* >1.5; gap contour areas where all of the units have indices *vj* < 1.5 are shaded in blue. Unshaded areas represent locations where counts are arranged effectively at random. SADIE software also provides information to plot other graphical displays such as “vector flow” plots and “empirical distribution function plots of ranked average outflow/inflow distances” (EDF) (Perry et al., 1999), although these are less represented in applied studies.

A very useful extension of SADIE measures the spatial association between patterns for two sets of count data, when they are sampled at the same spatially-referenced units (Perry and Dixon, 2002). This method is based on the correlation between the cluster indices of the two data sets at each sample unit, which is used to obtain an overall index of association (*X*), calculated as a mean of the local clustering index (*vi* or *vj*) of each sample unit. The *X* index describes the degree of association or dissociation between the two populations; these also provide tests (*P*) (Dutilleul, 1993) that may be mapped. In cases when the clustering indices of two populations compared at the same sampling site are both *vi* or both *vj*, the SADIE program assigns a positive index, indicating a local association for the sample site. Conversely, if the index of one population is *vi* and the other is *vj*, then the SADIE index will be negative, indicative of local dissociation. This test is a useful tool to identify interspecific interactions or responses to environmental heterogeneity (Perry and Dixon, 2002). A temporal sequence of “red-blue plots” (for a single set of data) and SADIE maps of local association (for two sets of data), can also provide information about temporal changes, when the same species was/were sampled at the same sample units on successive occasions.

### SADIE limitations and alternatives

Although SADIE is used increasingly for agricultural studies (see examples below), the methodology has some drawbacks. One of these is the analysis of dispersal of individuals from a single focus (*P*). To solve this problem, Korie et al. (1998) suggested the use of other statistics that allow the description of movement. Previously, for datasets in which spread was known to have originated from a central source, Perry (1995) used the SADIE (*Ia*) as well as the statistic δ defined as the distance between the centroid (*C*) (representing the average position of individuals in the experimental plot) and the known focus. Thus, δ is used to quantify the displacement of the entire organism population and represents the degree to which the counts occupy the edge rather than the center of the arrangement. To quantify the spread of the population about its displaced position, they defined the statistic Φ as the average of the squared distances of the position of each individual to the centroid (*C*). The statistic Δ was defined as the average distance of individual positions from the central release focus (*P*) and was used to quantify the total distance moved from P. Similarly, to characterize movement using aphid data, they used the statistics γ, to quantify the relative degree of movement between and within rows of the plot. When the insects moved entirely along a row, the value of γ would be 1; if their movements were entirely perpendicular to the rows, then γ would be 0, and if there was completely random movement the γ-value would be 0.5. These improved indices were used to successfully analyze dispersal from a single focus of different datasets for insects and plants (Korie et al., 1998; Smyrnioudis et al., 2001; Diaz et al., 2012). However, for datasets with unknown focus, which represent an invasion from the edge of a sample area, Korie et al. (1998) discussed the backtracking algorithm proposed by Perry (1995) to estimate the focus of a given cluster of individuals.

Another limitation detected in SADIE analysis discussed by Xu and Madden (2003) concerns the edge effect and the detection of small clusters in elongated areas. They demonstrated that this problem occurs because the local clustering indices (*vi* and *vj*) are mathematically related to the (*Ia*). A new method named MAPCOMP (MAP COMParison) was proposed by Lavigne et al. (2010) to analyse the spatial patterns of count data, and results were compared to those of the red-blue plot analysis of SADIE. MAPCOMP is based on permutation tests, as in the red-blue SADIE method, but uses the Hellinger distance between the density map of counts and the density map of sampling effort. This approach had better theoretical properties than the SADIE method to detect spatial heterogeneity when clusters were located on square or elongated domains and more or less close to the edges. Also, it is able to detect cluster patterns for small samples size and clusters with small radius. These advantages of MAPCOMP were demonstrated by Lavigne et al. (2010) in an analysis of the spatial distribution of codling moth, *Cydia pomonella*, diapausing larvae in eight orchards in France, suggesting that this method could be useful in cases of conservation biology of rare species or for agricultural pests where population densities are expected to be low and habitats may be geometrically intricate. However, further studies are needed to compare these new indices with conventional ones for different types of data.

### SADIE contributions to the spatial analysis of agroecosystems

The methods developed for SADIE are increasingly used to describe and understand many ecological processes. However, as shown by Perry et al. (2002), no single analytical method can identify all the spatial characteristics of data. Consequently, the use and comparison of more than one method for spatial analysis of ecological data is recommended.

Winder et al. (2001) provide the only report to date that used SADIE to analyze spatial patterns of both surface and subterranean organisms. Indeed, literature concerning belowground patterns is scare compared to that concerning aboveground spatial structure. For example, aboveground studies using SADIE have analyzed the spatial patterns of vegetation (Maestre, 2003), seed banks (González-Andújar et al., 1999), epidemics (Moreno et al., 2007), and various taxa of invertebrates (Winder et al., 1999; Rossi, 2003; Archard et al., 2004), to address theoretical and applied questions regarding agroecosystems. The studies to date primarily provide an understanding of interspecific interactions or responses to environmental heterogeneity (Perry and Dixon, 2002; Diaz et al., 2010; Nachappa et al., 2011). Several studies have employed SADIE methodology to enhance the manipulation of multiple natural enemies in ephemeral habitats, mainly in annual crops, which are characterized by temporal and spatial discontinuity for herbivorous pests and their natural enemies that exploit such habitats. Diaz et al. (2010) reported that the spatial-temporal congruence between two natural enemies, the fungus *Pandora neoaphidis* infecting aphids and the aphidophagous hoverflies larvae only occurred near to the time of lettuce harvest, reducing the risk of intraguild predation between these two natural enemies at field scale. As a consequence, both natural enemies could be good candidates to control aphids on lettuce in a conservation biological control strategy as part of an integrated pest management approach. In addition, this study highlighted the encouraging early temporal congruence of both natural enemies and their aphid prey through manipulation of external habitats that can provide refuge for both *P. neoaphidis* and hoverflies (Diaz et al., 2010). SADIE was used by Costamagna and Landis (2011) to demonstrate that the naturally occurring community of generalist predators exerts strong top–down suppression of *Aphis glycines* populations at multiple scales, and no evidence was found that the presence of prey refugia at these scales can lead to population outbreaks. Another recent example is the explicit use of SADIE spatial maps to investigate benefits from adjacent woody vegetation on predators and parasitoids within vineyards, which concluded with recommendations for management of the non-crop areas adjacent to farms (Thomson and Hoffmann, 2013).

**Figure 2:**
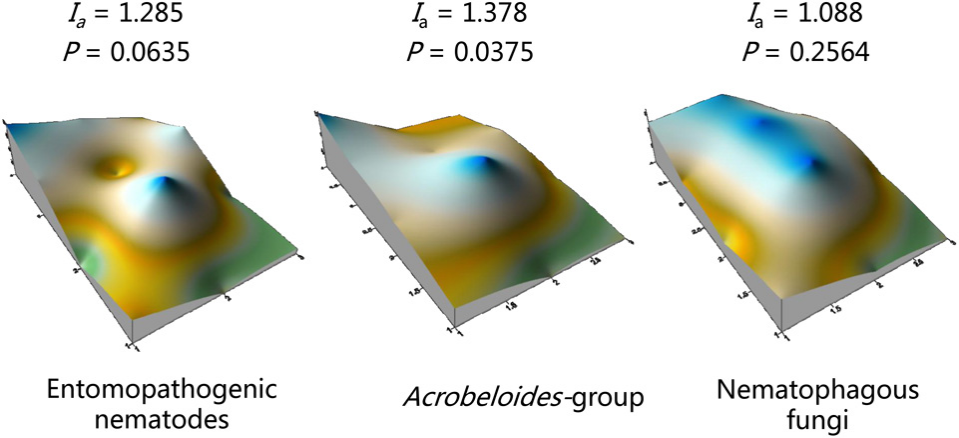
**Spatial patterns in a 10-ha citrus orchard surveyed in April 2009 using real time qPCR detection for entomopathogenic nematodes, free-living nematodes from the *Acrobeloides*-group, and nematophagous fungi**. SADIE aggregation indices (*Ia*) and probabilities that the counts are not randomly distributed are shown above figures. Reprinted from “Wide interguild relationships among entomopathogenic and free-living nematodes in soil as measured by real time qPCR (Campos-Herrera et al., 2012).

We find fewer examples of the use of SADIE to study belowground organisms, although several authors have employed it to characterize the spatial distribution of entomopathogenic nematodes. Wilson et al. (2003) used the spatial information provided by SADIE to study the persistence of the EPN species *Heterorhabditis bacteriophora*, when this nematode was used as an inundative biocontrol agent to control several species of scarab beetle larvae in crops. In this study, nematodes were applied following a uniform distribution, in one central circular patch and in individual patches. They observed that nematodes applied in patches moved from their initial application sites and became more evenly distributed, whereas the distribution of nematodes in plots with uniform application became patchier as nematodes died. Neither application method affected the persistence or efficacy of the nematodes. An elegant study by Spiridonov et al. (2007) identified aggregated and highly associated spatial patterns of *Steinernema feltiae* and *Steinernema affine* using Lloyd's index of patchiness and SADIE, when both species were measured at a fine (5 × 5 cm) scale. They characterized infective juvenile (IJ) physiological age (I–IV groups) based on the retencion of the sheath and a visible lipid content. Lloyds index identify group I as being the most aggregated and SADIE identify group II as the most aggregated. Both methods revealed random distribution for older individuals (groups III and IV) A discrepancy between the two aggregation indices when considering larval age groups was likely due to sampling scale, reinforcing the importance of using multiple methods to study spatial patterns. More recently, SADIE analysis was used to characterize the spatial patterns of entomopathogenic nematodes and other members of their soil food web such as NF and some free-living nematodes that compete with EPN for the cadaver (Figure 2) (Campos-Herrera et al., 2012). Positive correlations between the three guilds supported Lindford's hypothesis that introducing organic matter to soil (EPN-killed insects) promotes population growth by free living nematodes and natural enemies of nematodes (Linford et al., 1938).

Earthworms are recognized as soil engineers (Jones et al., 2006c) because the population or community distributions of these macroinvertebrates affect many ecosystem processes related to organic matter mineralization, aggregate formation (Richard et al., 2012) and resource availability in the soil (Jiménez et al., 2011). Spatial structure in earthworm communities has been related mainly to environmental heterogeneity posed by soil and vegetation patchiness and to environmental gradients at larger scale (Decaëns et al., 2009; Jiménez et al., 2011). Hernández et al. (2007) used geostatistical and multivariate analysis to explain the relationships between the horizontal distribution of earthworm communities and some soil factors in grassland in Madrid Spain. Jiménez et al. (2011) combined SADIE analysis to detect gap and patch clusters and association/dissociation between earthworm species with geostatistics, to investigate the spatial distribution of an earthworm community together with the heterogeneity of selected soil properties in a gallery forest of the Colombian “Llanos.” The degree of autocorrelation of spatial pattern was assessed with semi-variogram and they used the partial Mantel test to explain the relationship between the spatial pattern of earthworm density and soil environmental variables, concluding that the earthworm community of this gallery forest showed a random structure in a spatially clumped soil environment. Another study using SADIE provided insights into earthworm assembly rules on pastures of Northwestern France, by showing evidences for the driving role of local factors on earthworm spatial distribution and community assembly (Richard et al., 2012).

Multiyear and multilocation experiments used SADIE to study the corn rootworm *Diabrotica* spp. (Coleoptera: Chrysomelidae) to determine its spatial pattern and investigate spatial associations with environment factors in cornfields which provided an understanding of such spatial interrelationships has a potential to reduce sampling and management costs to reduce rootworm populations (Park and Tollefson, 2006). A recent study revealed the belowground distribution of *Diabrotica virgifera virgifera* using SADIE, demonstrating that larvae had an aggregated distribution throughout their development and that pattern changes of feeding larvae were linked with the feeding preference of the three larval instars for different types of roots (Schumann and Vidal, 2012).

The soil environment also constitutes an important reservoir for the diversity of entomopathogenic fungi, belonging to the orders Hypocreales and Entomophthorales, which are able to cause natural epizootics, regulating populations of above and belowground insects. The comparision of SADIE and Geographical Information System (GIS) was used to evaluate the validity of GIS to describe the spatial patterns of several soil borne entomopathogenic fungi, such as *Beauveria bassiana* and *Paecylomyces fumosoroseus* within agriculture and hedgerow ecosystems, respectively (Meyling and Eilenberg, 2006). They demonstrated the suitability of GIS for identifying distribution patterns of soil borne entomopathogenic fungi and the importance of large sample sizes to describe local biodiversity of the fungi in the soil environment.

The use and application of these statistical tools are increasing in scope, with tremendous potential to link above with belowground systems. In this regard, an interesting recent study developed by Eisenhauer et al. (2011) linked the impact of plant diversity and the presence above-belowground invertebrates to the stability of plant community productivity in space and time in experimental grassland communities. They concluded that changes in plant diversity at one trophic level are not reflected by changes in multitrophic interrelationships and that both above- and belowground invertebrates decouple the positive relationship between spatial and temporal stability of plant community productivity.

## Use of new methods to study subterranean biological control: case studies in florida

**Figure 3:**
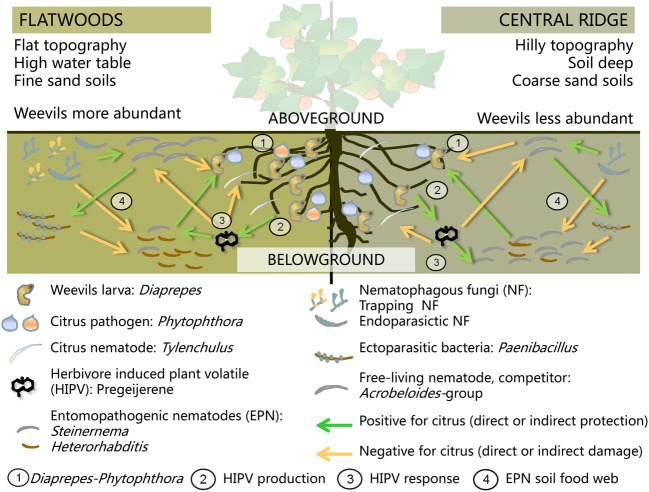
**Scheme of the belowground interactions among organisms on the citrus groves from Florida and their possible positive (green arrows) or negative (orange arrows) impact on the citrus production and health**. Selected trophic groups are represented: herbivore, citrus pathogens, plant-parasitic, entomopathogenic and free-living nematodes, nematophafous fungi, and ectoparasitic bacteria. Differences in number of individual and species composition are represented for two eco-region, central ridge, and fatwoods (see correspondence with colors and numbers in each part of the scheme). Production of the herbivore induced-plant volatiles (HIPV) is also represented. The trophic activities and interactions represented in this scheme are the following: (1) Synergic negative effect of *Diaprepes-Phytophthora* damage to roots; (2) Response of the citrus roots to *Diaprepes*-herbivore attack by producing the HIPV Pregeijerene; (3) Response of the soil organisms to the HIPV; (4) Trophic interactions among different soil organism. For further details, please, see details described in the section use of new methods to study subterranean biological control: case studies in Florida.

The measurement and analytical methods described in this paper were used in recent studies (Duncan and Stelinski, 2013) designed to help manage a subterranean weevil herbivore in Florida citrus orchards (Figure 3). *Diaprepes abbreviatus* is a serious citrus pest whose larvae cause feeding damage to the tree roots and facilitate root infection by plant pathogenic *Phytophthora* spp. oomyctes (Figure 3, interaction 1) (Graham et al., 2003; Dolinski et al., 2012). Duncan et al. (2003) suggested the possible involvement of natural enemies in the *D. abbreviatus* spatial pattern across different eco-regions of the Florida peninsula. Because weevils are more abundant in flatwoods regions than on the central ridge, they hypothesized that EPN species diversity, richness or abundance might be higher in the central ridge. Duncan et al. (2007) used conventional methods to characterize multitrophic links involved in the plant-herbivore-EPN food web in a Florida citrus orchard. In order to improve the ability to study relationships among weevils, EPNs, natural enemies of EPNs and abiotic soil conditions across the regions, molecular primers, and probes were developed to quantify selected soil organisms at multiple trophic levels from a single sample of DNA (Figure 3, interacion 4). Real time qPCR was then used to identify and quantify (1) 13 species of EPN (Campos-Herrera et al., 2011a,b, 2012, 2013), (2) 7 NF (Atkins et al., 2005; Zhang et al., 2006; Pathak et al., 2012), (3) 2 ectophoretic bacteria in the genus *Paenibacillus* that limit the mobility of the EPN infective juveniles (IJs) (Campos-Herrera et al., 2011b), (4) *Acrobeloides*-group of free-living bacteriophagous nematodes (FLBNs), reported to be competitors of EPNs in the cadaver (Campos-Herrera et al., 2012), and (v) a citrus pathogen associated with *D. abbreviatus* root damage (Huang et al., 2010).

Nematodes were extracted from soil in 53 orchards in a geospatial survey across the two regions. DNA from the nematode samples was then probed to quantify EPNs and organisms associated with nematodes in each sample. Spatial patterns from the data and relationships between patterns were then characterized using redundancy (RDA) and SADIE analyses (Campos-Herrera et al., 2013). A preliminary study to validate the methods from samples in a single orchard revealed significant aggregation of EPNs and of FLBNs, but not NF as measured by SADIE, and significant associations between all three guilds when measured by correlation analysis (Figure 2; Campos-Herrera et al., 2012). Across the two regions of the geospatial survey, three of the four most frequently encountered EPN species were significantly aggregated as measured by the SADIE *Ia* (Campos-Herrera et al., 2013). The spatial patterns of *Steinernema diaprepesi* and *Heterorhabditis zealandica* were significantly associated with that of the central ridge eco-region, supporting the possibility that EPNs influence the *D. abbreviatus* spatial pattern, because *S. diaprepesi* is reported to be more virulent to this weevil than other native EPN species (El-Borai et al., 2012). Variables that might affect EPN patterns were identified from redundancy analyses. Several variables that affect soil water potential (depth to groundwater, water holding capacity, content of clay and organic matter) were significant predictors of the soil community composition. These variables explained more than 40% of the variability of *S. diaprepesi* in the survey and suggested that the nematode is encountered most frequently and in greatest abundance in soil with lower rather than higher water potential. A long-term field experiment provided some support for the possibility that native EPNs regulate weevils more effectively in drier soil conditions. Duncan et al. (2013) substituted sand for native soil in tree planting holes. Fewer weevils emerged from sand and trees grew larger than in the native loamy sand soil. Sentinel weevil larvae buried in the plots were killed at higher rates than in native soil; however, the abundance of EPNs measured by qPCR was not different in either soil. Thus, greater regulation of weevils by native EPNs in soil with low water potential may result because such soils favor parasitism by some species, rather than by increasing EPN abundance.

A semiochemical basis for multitrophic relationships (citrus plant-herbivore-biological control agent-natural enemies) has also been studied (Ali et al., 2010, 2011, 2012, 2013). Molecular probes were used together with *in situ* recovery of HIPV to demonstrate that naturally occurring EPNs as well as free living, bactivorous nematodes that sometimes compete with EPNs were attracted by the volatile compound pregeijerene (1,5-dimethylcyclodeca-1,5,7-triene) that emanates from citrus roots fed upon by weevils (Ali et al., 2012, 2013) (Figure 3, interactions 2 and 3). The net effect of the FLBNs on EPN efficacy in this system is unknown and ultimately the effects of these interactions on plant production/yield need to be measured to understand whether manipulating indirect plant defenses is practical for sustainable pest control.

## Future prospects

Rapidly evolving techniques to measure and analyze spatial and temporal dynamics of community structure, interguild relationships, and plant communication present ever greater opportunities for interdisciplinary studies to fundamentally advance our understanding of how plants interact with other organisms and their environment. For example, stable isotope analysis continues to be widely used to assess prey-predator links and ecosystem functioning such as feedback in prey availability and predator hunting style (Wimp et al., 2013). However, molecular techniques such as those described here are also helping to reveal and characterize trophic interactions between many organisms, such as predation rates in the field by various species of NF (Pathak et al., 2012) or the plant preferences of generalist vs. specialist herbivores and their effects on the natural resources (Hereward and Walter, 2012). A method to analyze invertebrate regurgitate, made possible by refined PCR techniques, has eliminated the need to destructively sample rare specimens for predator-prey assessment (Waldner and Traugott, 2012). The development of multiplex systems will further encourage the use of PCR methods to provide new insights into predator-prey relationships that serve to develop new approaches for biological control of pests. Linking indirect plant defense and predator-prey behavior, in time and space, by using PCR-methods to identify and quantify organisms and their relationships with plant signaling has obvious potential for pest management. We have described reports of this phenomenon for EPNs in citrus orchards and maize fields. PCR was also recently used to identify important arthropod and acarid predators of *D. virgifera virgifera* (Lundgren et al., 2009; Lundgren and Fergen, 2011). Linking knowledge of how maize semiochemicals function to affect a diverse predator/pathogen guild would be an important step toward a holistic understanding of food web dynamics and how they can be manipulated to enhance sustainable pest management. Clearly, the daunting challenges to characterizing the functions of soil communities posed by their taxonomic and behavioral complexity are being overcome by rapid methodological advances on many fronts.

### Conflict of interest statement

The authors declare that the research was conducted in the absence of any commercial or financial relationships that could be construed as a potential conflict of interest.
